# Asynchronous quasi delay insensitive majority voters corresponding to quintuple modular redundancy for mission/safety-critical applications

**DOI:** 10.1371/journal.pone.0239395

**Published:** 2020-09-22

**Authors:** P. Balasubramanian, N. E. Mastorakis

**Affiliations:** 1 School of Computer Science and Engineering, Nanyang Technological University, Singapore, Singapore; 2 Department of Industrial Engineering, Technical University of Sofia, Sofia, Bulgaria; University of Scranton, UNITED STATES

## Abstract

Electronic circuits and systems employed in mission- and safety-critical applications such as space, aerospace, nuclear plants etc. tend to suffer from multiple faults due to radiation and other harsh external phenomena. To overcome single or multiple faults from affecting electronic circuits and systems, progressive module redundancy (PMR) has been suggested as a potential solution that recommends the use of different levels of redundancy for the vulnerable portions of a circuit or system depending upon their criticality. According to PMR, triple modular redundancy (TMR) can be used where a single fault is likely to occur and should be masked, and quintuple modular redundancy (QMR) can be used where double faults are likely to occur and should be masked. In this article, we present asynchronous QDI majority voter designs for QMR and state which are preferable from cycle time (i.e., speed), area, power, and energy perspectives. Towards this, we implemented example QMR circuits in a robust QDI asynchronous design style by employing a delay insensitive dual rail code for data encoding and adopting four-phase handshake protocols for data communication. Based on physical implementations using a 32/28nm CMOS process, we find that our proposed QMR majority voter achieves improved optimization in speed and energy.

## 1. Introduction

Electronic circuits/systems used in mission-critical applications such as space, and safety-critical applications such as aerospace, nuclear power plants, electric power transmission and distribution, and industrial control automation etc. usually incorporate some form of N-modular redundancy (NMR) to overcome bounded faults whose occurrences may be temporary or permanent in nature. In NMR, a function unit and (N–1) identical copies of the function unit, where N is odd, are used, where a function unit may be a circuit or a sub-system or a system. In NMR, majority of the function units is required to maintain the correct operation. In other words, at least (N+1)/2 function units should operate correctly meaning that the faults of (N–1)/2 function units would be masked. A function unit may produce one or more outputs. If each function unit in an NMR implementation produces M outputs, the respective outputs of all the function units are connected to M N-input majority voters, which in turn produce the primary outputs based on the Boolean majority [1]. Hence, N identical function units and N-input majority voters are used to realize an NMR implementation.

The majority voter, although being an important decision making element, usually forms a small component of a circuit/system used in a mission-/safety-critical application. Hence, the majority voter is generally assumed to be perfect. However, if there may arise a concern about the majority voter that it may become a single point of failure then the majority voter may be duplicated like the function units [2]. This implies that instead of having one majority voter providing the input for the next circuit/system stage, identical majority voters may be used and the outputs of these majority voters can provide similar inputs for the next circuit/system stage. This kind of implementation involving duplication of majority voters avoids the likelihood of a single point of failure [3]. For example, in the Saturn launch vehicle digital computer the majority voters were triplicated to avoid a single point of failure [4]. Another alternative is to radiation harden the entire circuit/system at the process level by considering advanced processes based on the silicon-on-insulator (SOI) technology such as a fully or a partially depleted SOI for physical realization [5].

Triple modular redundancy (TMR) is the basic and the most widely used NMR scheme. In TMR, three identical function units are used whose outputs are connected to three-input majority voters and the temporary or permanent fault of an arbitrary function unit would be masked. Thus, TMR can efficiently withstand a single upset (fault). To overcome double upsets, quintuple modular redundancy (QMR), which is a higher order NMR, can be adopted where five identical function units are used whose outputs are connected to five-input majority voter(s) and the temporary or permanent faults of two arbitrary function units would be masked. Other higher order NMR schemes involving seven or more function units are also realizable although they are sparingly used.

It was noted in [6] that multiple bit upsets are likely to occur in combinational and sequential digital circuits. In [7], an investigation was carried out to find the proportion of single and multiple bit upsets that electronic devices such as field programmable gate arrays may encounter in a realistic space environment. It was found that a big majority of the upsets are single upsets while a small minority are double upsets. The percentages of triple and quadruple upsets were found to be very small. Hence, adopting TMR and QMR might suffice for a mission- or safety-critical application where TMR can be used to overcome single upsets and QMR can be used to overcome double upsets.

In [8], progressive module redundancy (PMR) was suggested as a fault-tolerant design strategy which recommends the use of different levels of redundancy for the critical portions of a circuit or system depending on their vulnerability. For example, TMR can be deployed in those portions where single upsets are likely to occur and QMR can be deployed in those portions where double upsets are likely to occur. In a way, the technique of selective insertion of TMR suggested in [9] is extended to include the selective insertion of QMR in the PMR scheme.

In any NMR implementation, the majority voter is indispensable. Many synchronous majority voters for TMR [10–12] and few synchronous majority voters for QMR [13–15] have been presented in the literature. An asynchronous majority voter for TMR corresponding to a bundled-data handshake protocol was also presented in the literature [16]. However, in asynchronous design, the bundled-data handshake protocol is known to be less robust, and the four phase handshake protocol used in a quasi delay insensitive (QDI) asynchronous design is understood to be more robust. Recently, robust QDI asynchronous majority voters for TMR were presented [17, 18]. In this work, we discuss and propose QDI majority voters for QMR. To our knowledge, this is the first work that deals with QDI asynchronous majority voters for QMR implementations.

The rest of this article is organized into four sections. Section 2 provides a background about QDI logic design. Section 3 presents the various QDI majority voters for QMR. Section 4 presents the simulation results for the example QMR circuit implementations which utilize the majority voters to be discussed. Screenshots of a portion of the simulation waveforms corresponding to the proposed QMR majority voter are also given in Section 4. Finally, we draw some conclusions in Section 5.

## 2. QDI asynchronous logic design

The consideration of a QDI asynchronous logic design is motivated by important factors such as innate resilience to electromagnetic interference [19], inherent tolerance to parametric variations [20] and harsh environmental phenomena [21], low power operation [22], and natural resistance to power analysis attacks [23], all of which are relevant for mission- and safety-critical applications.

QDI logic design is the practically realizable delay insensitive (DI) design which employs a DI code [24] for data encoding and four-phase return-to-zero (RTZ) [25] or return-to-one (RTO) [26] handshake protocols for data communication. However, the main difference between a DI design and a QDI design is that the latter incorporates the isochronic fork assumption [27], which represents the weakest compromise to delay insensitivity. An isochronic fork basically refers to a signal node or a junction from where if two or more wire branches emerge, the timing assumption is that any signal transition occurring on an isochronic fork, whether it be binary 0 to binary 1 or binary 1 to binary 0, it is assumed to happen concurrently on all the wire branches arising out of that fork. It is noted in [27] that without the isochronic fork assumption, DI circuits cannot be realized in reality. It is observed in [28] that the isochronic fork assumption is actually a mild timing assumption which helps to increase the computational power of pure DI circuits. Further, it has been shown in [29] that the isochronic fork assumption is realizable even in nanoscale design geometries.

### 2.1. QDI circuit stage, data encoding, and four-phase handshaking

**Fig 1:**
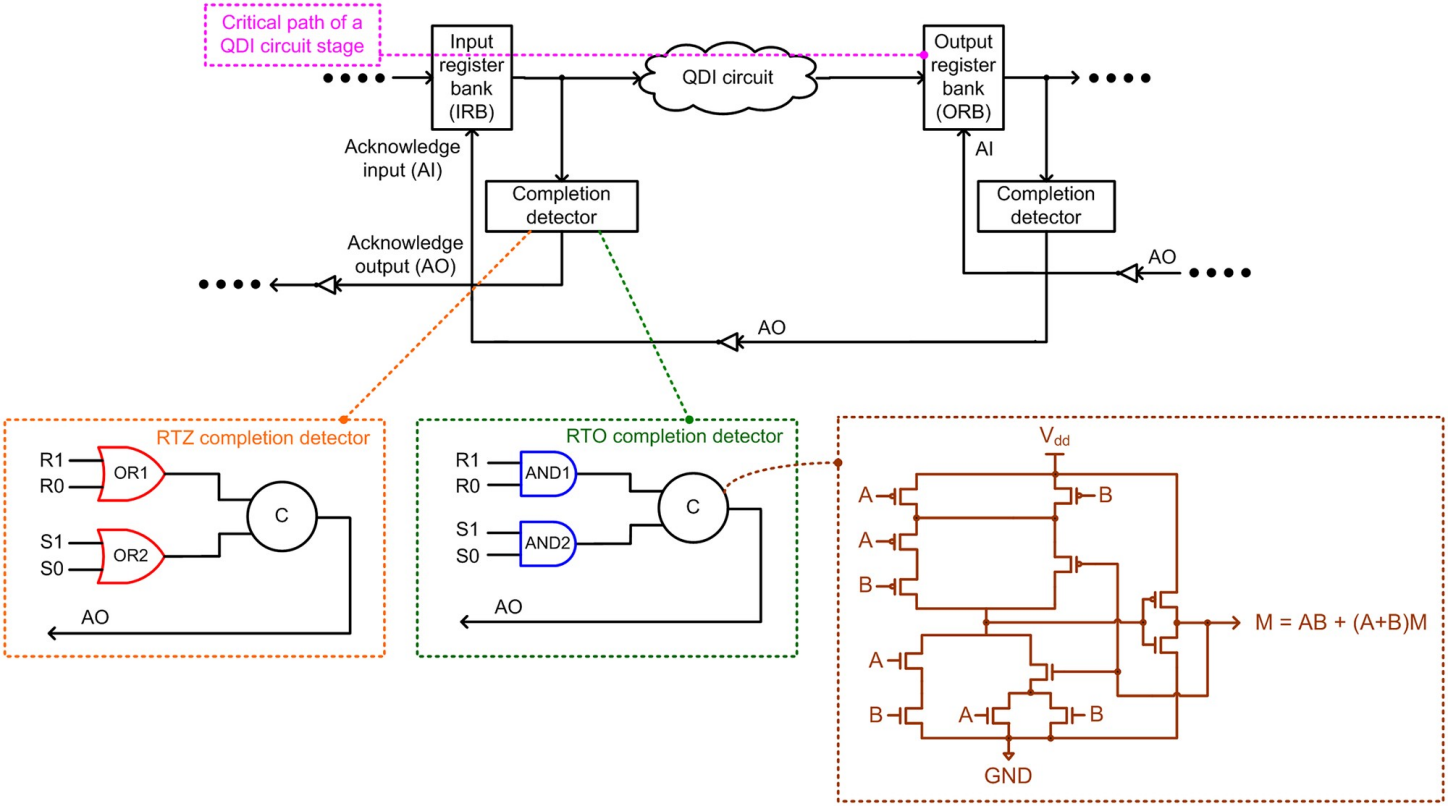
A typical quasi delay insensitive (QDI) asynchronous circuit stage.

The typical block schematic of a QDI circuit stage comprising input and output registers is shown in Fig 1, which consists of an input register bank (IRB), an output register bank (ORB), a QDI circuit that is sandwiched between these register banks, and completion detectors. Acknowledgment input (AI) and acknowledgment output (AO) signals are exchanged between the IRB and ORB, and the data or spacer is input to a QDI circuit through the IRB based on the states of AI and AO signals which are also called handshake signals. AI is the Boolean complement of AO, and vice-versa. A typical QDI circuit stage consists of an IRB, a completion detector, and a QDI circuit. In fact, the ORB serves as the IRB for a subsequent circuit stage. The critical data path encountered in a QDI circuit stage is highlighted by the dotted pink line in Fig 1.

The IRB and ORB consist of a series of registers, which are basically 2-input C-elements. For example, in the IRB, a 2-input C-element is allotted for each rail of a dual rail encoded input. The C-element [30] produces 1 if all its inputs are 1 and produces 0 if all its inputs are 0. If any input is different from the rest of the inputs, the C-element would retain its existing state. The transistor level static CMOS realization of a 2-input C-element is shown in Fig 1 [17] within the dotted brown box, where A and B are the inputs and M is the output.

The inputs and outputs of a QDI circuit are encoded using a DI code. Among the family of DI codes [24], the dual rail code is the simplest member which has been widely used for QDI circuit designs [25]. In Fig 1, (R1, R0) and (S1, S0) represent the example dual rail encoded equivalents of the single rail inputs R and S respectively. According to dual rail encoding and RTZ handshaking, an input R is encoded as (R1, R0) where R = 1 is encoded as R1 = 1 and R0 = 0, and R = 0 is encoded as R0 = 1 and R1 = 0. These assignments are called ‘data’. R1 = R0 = 0 is the ‘spacer’, and R1 = R0 = 1 is considered indeterminate. In the case of RTZ handshaking, binary 1 on one of the encoded dual rails is used to represent the data. Hence, the signal transitions in a QDI circuit will be monotonically increasing (i.e., 0 to 1) for the application of data, and monotonically decreasing (i.e., 1 to 0) for the application of the spacer [31].

On the other hand, according to dual rail encoding and RTO handshaking, an input R is encoded as (R1, R0) where R = 1 is encoded as R1 = 0 and R0 = 1, and R = 0 is encoded as R0 = 0 and R1 = 1. These two assignments are called ‘data’. R1 = R0 = 1 is the ‘spacer’, and R1 = R0 = 0 is considered to be indeterminate. In the case of RTO handshaking, binary 0 on one of the encoded dual rails is used to represent the data. Thus, the signal transitions in a QDI circuit will be monotonically decreasing for the application of data, and monotonically increasing for the application of the spacer.

Handshaking is performed between IRB and ORB involving the QDI circuit, which is responsible for processing the data and the spacer. Four steps are involved in RTZ and RTO handshaking, and they are referred to as four-phase handshake protocols. The steps involved in RTZ and RTO handshaking are described in [17], and an interested reader may refer to the same for details. Here, it is sufficient to state that in the case of RTZ handshaking, the inputs are supplied conforming to the sequence of data, spacer, data, spacer and so on. In the case of RTO handshaking, the inputs are supplied conforming to the sequence of spacer, data, spacer, data and so on.

The gate level detail of the example completion detectors corresponding to RTZ and RTO handshaking is shown in Fig 1 inside the dotted orange and green boxes. The completion detector indicates i.e., acknowledges the receipt of all the primary inputs given to a QDI circuit. In the case of RTZ (RTO) handshaking, 2-input OR (AND) gates are used to combine the respective dual rails of all the encoded inputs, and the outputs of all such 2-input OR (AND) gates are combined using a C-element or a tree of C-elements to produce AO. The main difference between RTZ and RTO completion detectors is that excepting the C-elements which are retained along with their respective inputs, the OR gates in the RTZ completion detector are replaced by their gate duals viz. the AND gates to obtain the RTO completion detector.

### 2.2. Indicating circuits

**Fig 2:**
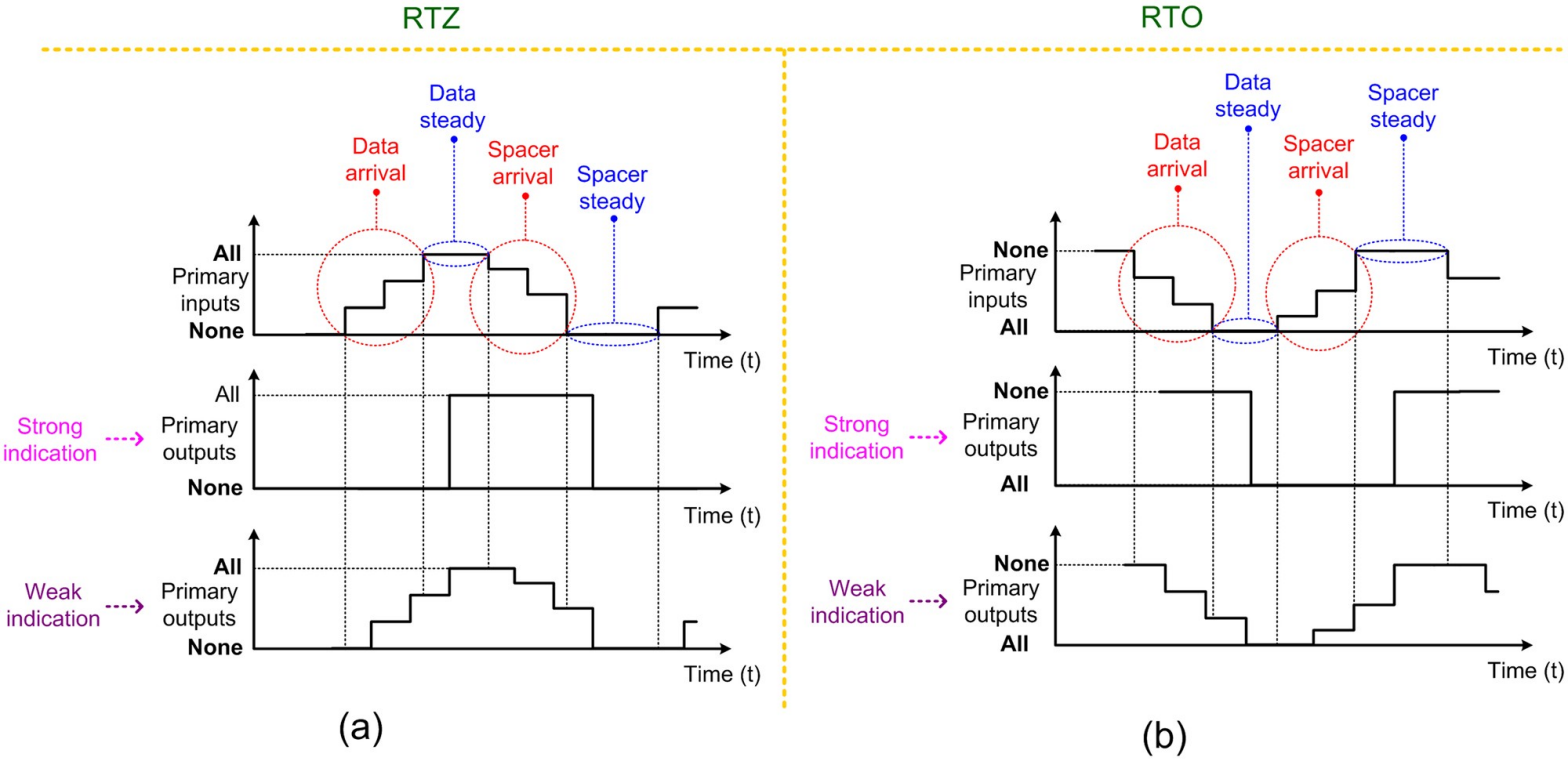
Timing behavior of indicating circuits with respect to: (a) RTZ; and (b) RTO handshaking.

There are two types of indicating QDI circuits namely strong indication and weak indication circuits [32]. The timing relation between the receipt of primary inputs and the production of primary outputs of strong indication and weak indication circuits is depicted by representative diagrams in Fig 2. Fig 2a corresponds to RTZ handshaking and Fig 2b corresponds to RTO handshaking.

Strong indication circuits wait to receive all the data and spacer, and after receiving them would process them to produce all the primary outputs. A strong indication circuit would produce a primary output only after receiving and processing all the primary inputs. Hence, *strong indication circuits may comprise one or more primary outputs*.

Weak indication circuits can process and produce some primary outputs after receiving some of the primary inputs. However, weak indication circuits would produce the last primary output only after receiving and processing the last primary input. Thus, a weak indication circuit requires at least two primary outputs so that even if one primary output is produced early after receiving and processing some of the primary inputs, the other primary output would be produced after receiving and processing the remaining primary inputs. Hence, *weak indication circuits require at least two primary outputs*. For example, a weak indication full adder [33] adding two input bits along with a carry input may generate/kill the carry output early if the two input bits are equal to 1/0 while the corresponding sum output would be produced only after the carry input is received and processed. Given this, since the QMR majority voter has five inputs and a single output, a weak indication realization is not feasible. Therefore, this work describes strong indication QMR majority voters.

## 3. QDI QMR majority voters

This section discusses many strong indication QMR majority voters including our proposed design.

### 3.1. Decomposed DIMS QMR majority voter

The delay insensitive minterm synthesis (DIMS) method [34] may be thought of as an extended version of the DI regular expression recognizer presented in [35]. The DIMS method basically describes a logic function in a canonical sum-of-products (CSOP) form. A CSOP form comprises product terms which are realized using all the literals constituting the support set of a function. Let us assume that (A1, A0), (B1, B0), (C1, C0), (D1, D0) and (E1, E0) represent the dual rail encoded primary inputs of a QMR majority voter, and (X1, X0) represents its dual rail encoded primary output. The truth table of the dual-rail encoded QMR majority voter that corresponds to RTZ handshaking is given in Table 1, and the canonical output SOP expressions are given by (1) and (2).

$$\begin{array}{l} \text{X}1=\text{A}0\text{B}0\text{C}1\text{D}1\text{E}1+\text{A}0\text{B}1\text{C}0\text{D}1\text{E}1+\text{A}0\text{B}1\text{C}1\text{D}0\text{E}1+\text{A}0\text{B}1\text{C}1\text{D}1\text{E}0+\\ \text{A}0\text{B}1\text{C}1\text{D}1\text{E}1+\text{A}1\text{B}0\text{C}0\text{D}1\text{E}1+\text{A}1\text{B}0\text{C}1\text{D}0\text{E}1+\text{A}1\text{B}0\text{C}1\text{D}1\text{E}0+\\ \text{A}1\text{B}0\text{C}1\text{D}1\text{E}1+\text{A}1\text{B}1\text{C}0\text{D}0\text{E}1+\text{A}1\text{B}1\text{C}0\text{D}1\text{E}0+\text{A}1\text{B}1\text{C}0\text{D}1\text{E}1+\\ \text{A}1\text{B}1\text{C}1\text{D}0\text{E}0+\text{A}1\text{B}1\text{C}1\text{D}0\text{E}1+\text{A}1\text{B}1\text{C}1\text{D}1\text{E}0+\text{A}1\text{B}1\text{C}1\text{D}1\text{E}1 \end{array}$$ (1)

$$\begin{array}{l} \text{X}0=\text{A}0\text{B}0\text{C}0\text{D}0\text{E}0+\text{A}0\text{B}0\text{C}0\text{D}0\text{E}1+\text{A}0\text{B}0\text{C}0\text{D}1\text{E}0+\text{A}0\text{B}0\text{C}0\text{D}1\text{E}1+\\ \text{A}0\text{B}0\text{C}1\text{D}0\text{E}0+\text{A}0\text{B}0\text{C}1\text{D}0\text{E}1+\text{A}0\text{B}0\text{C}1\text{D}1\text{E}0+\text{A}0\text{B}1\text{C}0\text{D}0\text{E}0+\\ \text{A}0\text{B}1\text{C}0\text{D}0\text{E}1+\text{A}0\text{B}1\text{C}0\text{D}1\text{E}0+\text{A}0\text{B}1\text{C}1\text{D}0\text{E}0+\text{A}1\text{B}0\text{C}0\text{D}0\text{E}0+\\ \text{A}1\text{B}0\text{C}0\text{D}0\text{E}1+\text{A}1\text{B}0\text{C}0\text{D}1\text{E}0+\text{A}1\text{B}0\text{C}1\text{D}0\text{E}0+\text{A}1\text{B}1\text{C}0\text{D}0\text{E}0 \end{array}$$ (2)

**Table 1:** Truth table of QDI QMR majority voter corresponding to RTZ handshaking.

Dual-rail encoded primary inputs										Dual-rail encoded primary output	
A1	A0	B1	B0	C1	C0	D1	D0	E1	E0	X1	X0
0	1	0	1	0	1	0	1	0	1	0	1
0	1	0	1	0	1	0	1	1	0	0	1
0	1	0	1	0	1	1	0	0	1	0	1
0	1	0	1	0	1	1	0	1	0	0	1
0	1	0	1	1	0	0	1	0	1	0	1
0	1	0	1	1	0	0	1	1	0	0	1
0	1	0	1	1	0	1	0	0	1	0	1
0	1	0	1	1	0	1	0	1	0	1	0
0	1	1	0	0	1	0	1	0	1	0	1
0	1	1	0	0	1	0	1	1	0	0	1
0	1	1	0	0	1	1	0	0	1	0	1
0	1	1	0	0	1	1	0	1	0	1	0
0	1	1	0	1	0	0	1	0	1	0	1
0	1	1	0	1	0	0	1	1	0	1	0
0	1	1	0	1	0	1	0	0	1	1	0
0	1	1	0	1	0	1	0	1	0	1	0
1	0	0	1	0	1	0	1	0	1	0	1
1	0	0	1	0	1	0	1	1	0	0	1
1	0	0	1	0	1	1	0	0	1	0	1
1	0	0	1	0	1	1	0	1	0	1	0
1	0	0	1	1	0	0	1	0	1	0	1
1	0	0	1	1	0	0	1	1	0	1	0
1	0	0	1	1	0	1	0	0	1	1	0
1	0	0	1	1	0	1	0	1	0	1	0
1	0	1	0	0	1	0	1	0	1	0	1
1	0	1	0	0	1	0	1	1	0	1	0
1	0	1	0	0	1	1	0	0	1	1	0
1	0	1	0	0	1	1	0	1	0	1	0
1	0	1	0	1	0	0	1	0	1	1	0
1	0	1	0	1	0	0	1	1	0	1	0
1	0	1	0	1	0	1	0	0	1	1	0
1	0	1	0	1	0	1	0	1	0	1	0

Eqs (1) and (2) are inherently in the disjoint sum of products (DSOP) form. The products in a DSOP expression are orthogonal to each other i.e., the logical conjunction of any two products in a DSOP form would yield 0 [36]. This implies that only one product term would be activated in a DSOP equation upon the application of an input data, which satisfies the monotonic cover constraint [25].

Eqs (1) and (2) when synthesized according to the DIMS approach would require thirty-two 5-input C-elements and ten 4-input OR gates. The C-element is usually not available in a standard digital cell library since it is an asynchronous gate and hence it should be custom designed. Moreover, there are fan-in limitations when designing a gate in a static CMOS style. Hence, a 2-input C-element was custom designed using a 32/28nm standard cell library [37] by incorporating feedback in an AO222 complex gate as shown in [17].

Since (1) and (2) should be implemented using 2-input C-elements first they should be safely decomposed to avoid any gate orphan. In QDI circuits, wire orphans and gate orphans may become problematic and so they should be carefully dealt with during the physical realization. Wire orphan refers to an unacknowledged signal transition on a primary input wire, and gate orphan refers to an unacknowledged signal transition on an intermediate gate output.

**Fig 3:**
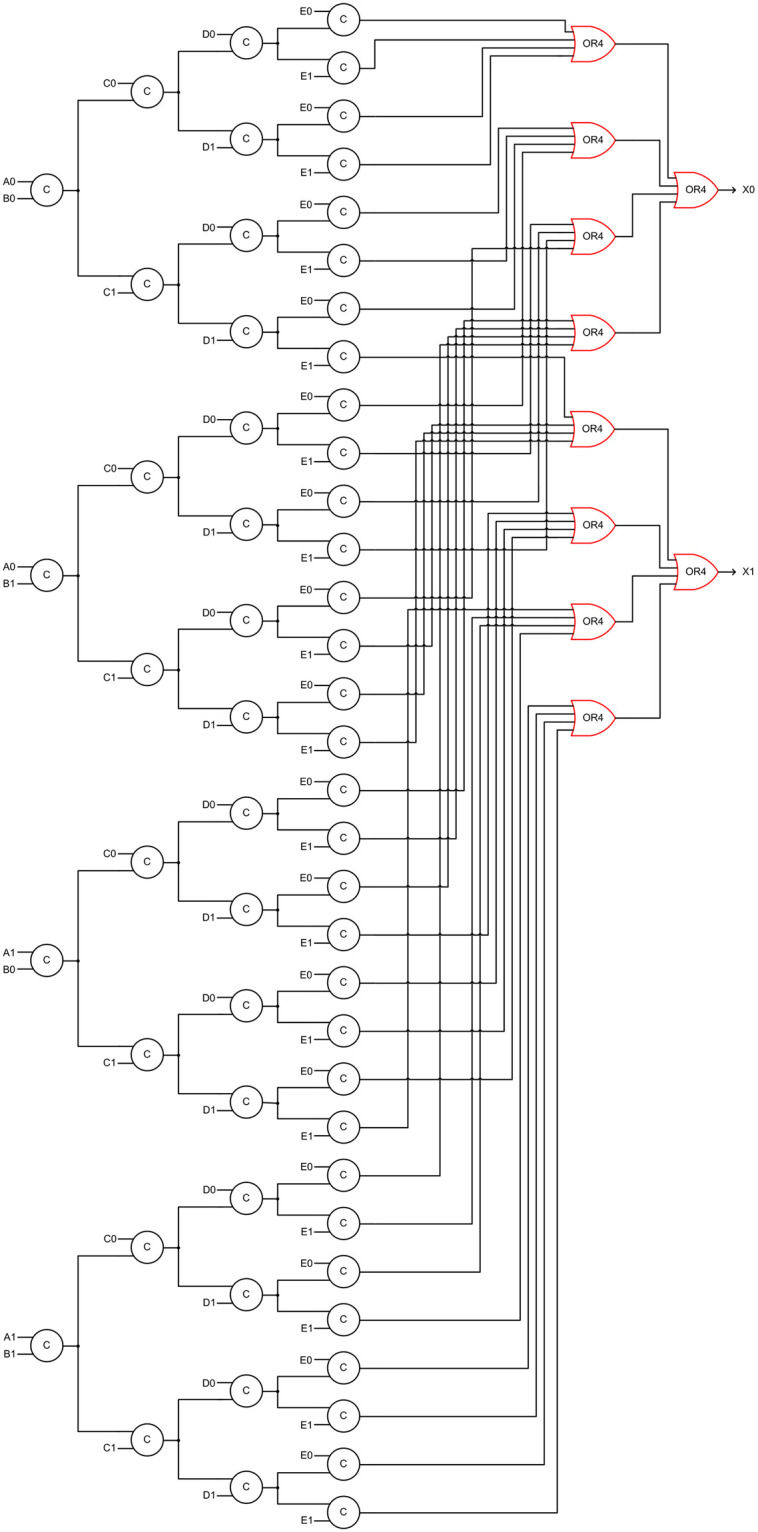
Safely decomposed DIMS QMR majority voter corresponding to RTZ handshaking. The OR gates shown in red should be replaced by AND gates to obtain the RTO equivalent circuit.

Wire and gate orphans have been explained through an example in [38], and an interested reader may refer to the same for details. Wire orphans are less problematic as they are easily overcome through the isochronic fork assumption when imposed on all the primary inputs, which is common in QDI circuits. This is because, referring to Fig 1, each primary input given to a QDI circuit is also given to a completion detector. Hence, a signal transition on a primary input appears concurrently across the QDI circuit and across the completion detector. When a signal transition appears on an intermediate gate output, and if it is not acknowledged by a similar signal transition in a subsequent gate output, then that unacknowledged signal transition is said to be a gate orphan. Gate orphans should be avoided in indicating circuits as they could affect the quasi delay insensitivity. Gate orphans can be avoided by adopting safe QDI logic decomposition techniques [39, 40]. The decomposed DIMS QMR majority voter realized through a safe QDI logic decomposition is shown in Fig 3, which corresponds to RTZ handshaking. To obtain the RTO equivalent circuit, the OR gates shown in red in Fig 3 should be replaced by their gate duals viz. the AND gates. In general, excepting the C-elements, each gate in a QDI circuit that corresponds to RTZ handshaking should be replaced by its respective gate dual to obtain the RTO equivalent circuit. This principle has already been proved in [41]. Henceforth, we shall refer to the decomposed DIMS QMR majority voter as ‘DDIMS-QMV’ for brevity.

### 3.2. Dysart logic based QMR majority voter

**Fig 4:**
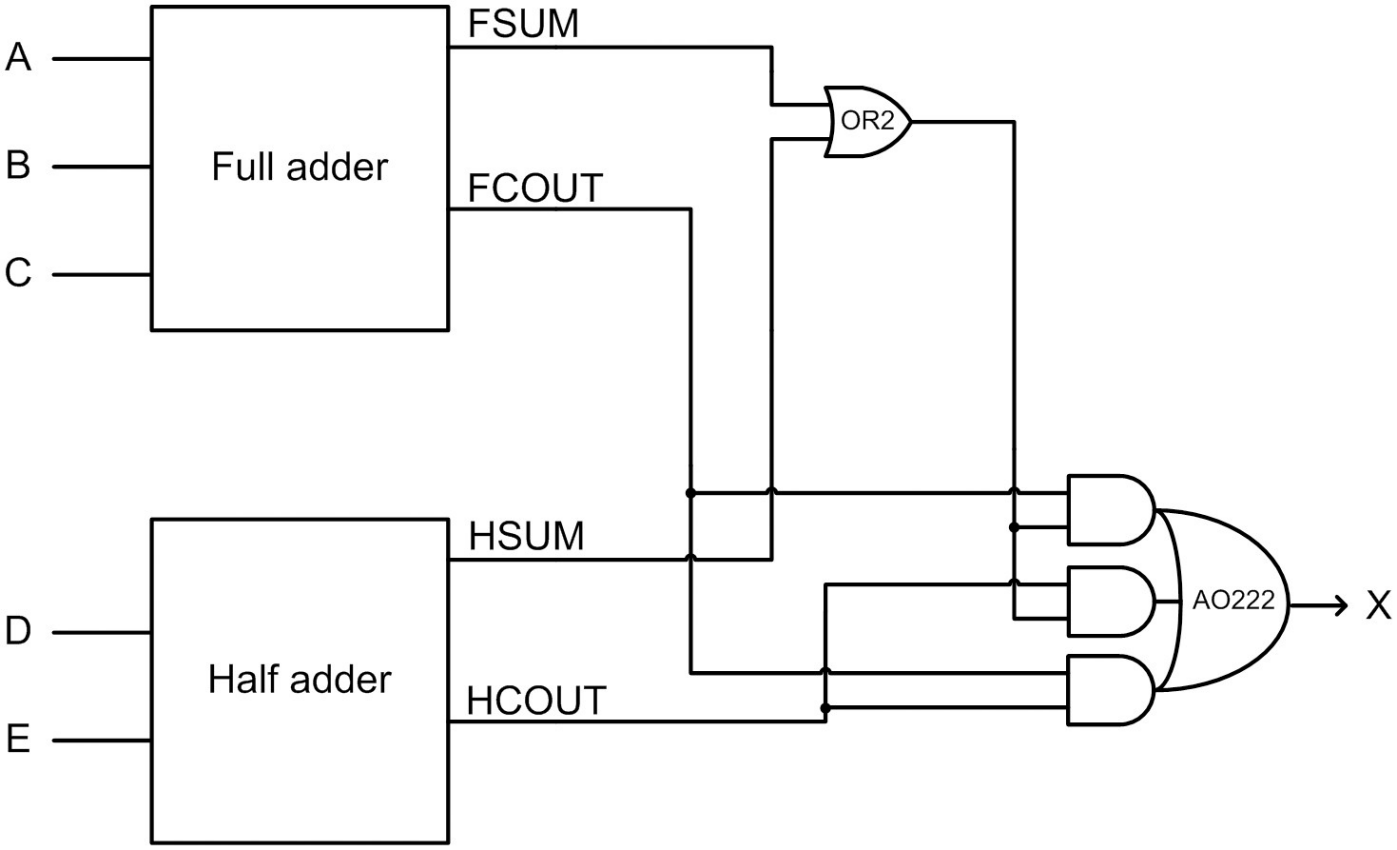
Dysart’s synchronous QMR majority voter.

Dysart [13] designed a synchronous QMR majority voter using a full adder, a half adder, and a couple of logic gates as shown below in Fig 4, where A, B, C, D and E represent the voter inputs and X represents the voter output. FSUM and FCOUT represent the sum and carry outputs of the full adder while HSUM and HCOUT represent the sum and carry outputs of the half adder in Fig 4.

**Fig 5:**
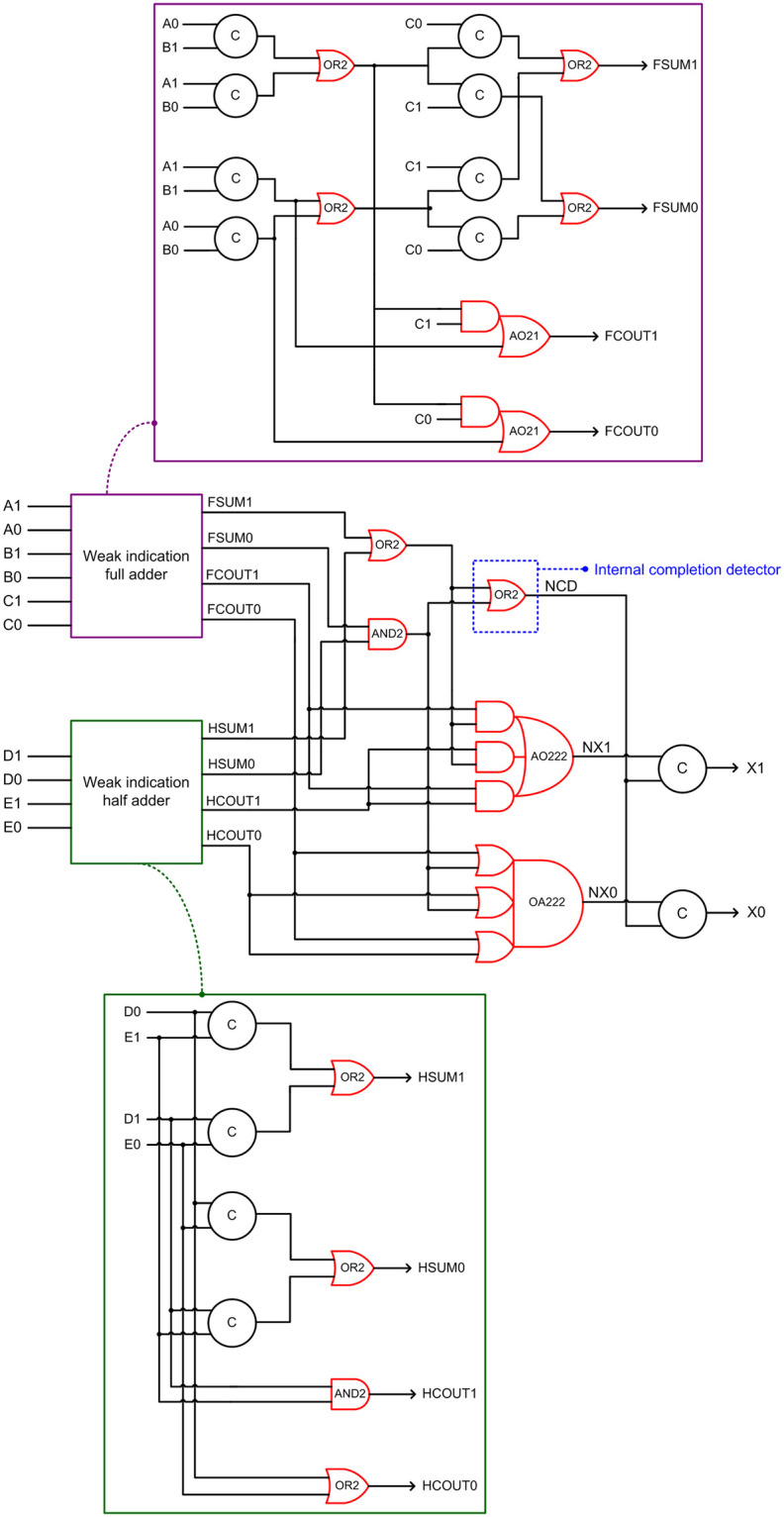
QDI asynchronous QMR majority voter corresponding to RTZ handshaking, realized based on Dysart’s synchronous QMR majority voter logic. The gates highlighted in red should be replaced by their respective gate duals to obtain the RTO equivalent circuit.

We have transformed the above synchronous design into a QDI asynchronous design by using the dual rail combinational logic (DRCL) design method of [42]. The resulting Dysart based QDI QMR majority voter corresponding to RTZ handshaking is shown in Fig 5, which shall be referred to as ‘Dysart-QMV’ henceforth for brevity. To realize the full adder and the half adder logic, the optimized weak indication full adder of [43] and the optimized weak indication half adder of [44] were used. The gate level details of the weak indication full adder and the weak indication half adder are shown in the violet and green boxes in Fig 5. It may be noted that the indication of the primary inputs of the majority voter is taken care of by the sum output logic of weak indication full and half adders. The carry outputs of the full adder and the half adder may be produced early whereas the sum outputs of the full adder and the half adder would be produced via strong indication. In Fig 5, the intermediate dual rail output (NX1, NX0) is logically equivalent to the voter’s dual rail primary output (X1, X0). An internal completion detector is introduced, which is shown within the dotted blue box in Fig 5, to ensure the completion of internal processing within the voter to avoid any gate orphan, and its output is denoted as NCD. NCD is synchronized with NX1 and NX0 using two 2-input C-elements to generate the majority voter’s primary output (X1, X0). To obtain the RTO equivalent of Dysart-QMV, all the gates highlighted in red in Fig 5 should be replaced by their respective gate duals i.e., the simple gates OR2 and AND2 should be replaced by AND2 and OR2 gates respectively and the complex gates AO21, AO222 and OA222 should be replaced by OA21, OA222 and AO222 respectively.

### 3.3. Simple and complex gates based QMR majority voter

**Fig 6:**
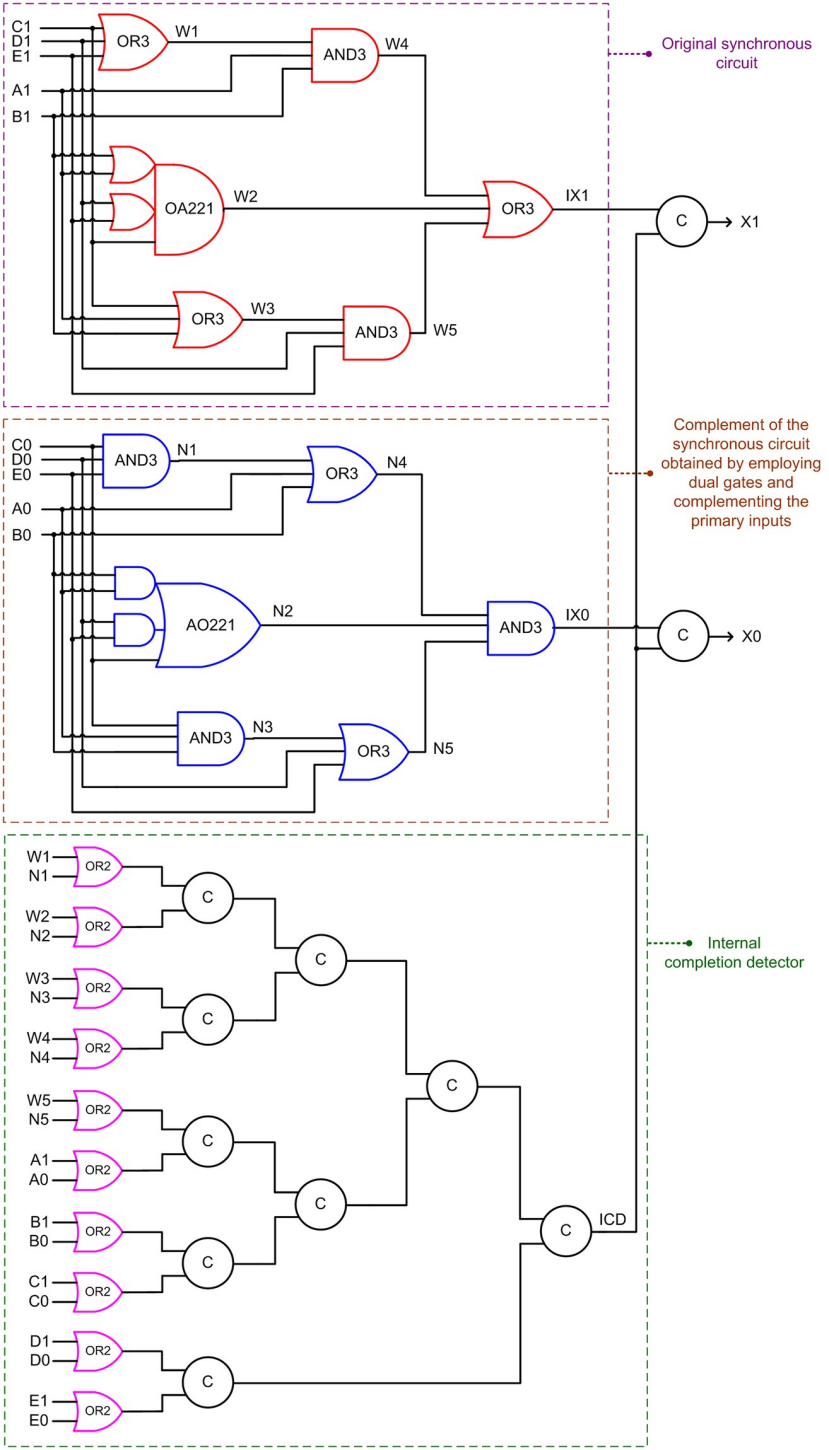
QDI QMR realization of a synchronous QMR majority voter [14], corresponding to RTZ handshaking. The gates highlighted in red, blue and pink should be replaced by their respective gate duals to obtain the RTO equivalent.

An optimized synchronous QMR majority voter consisting of simple and complex gates was presented in [14]. A QDI version of the same realized using the DRCL design method is shown below in Fig 6, which corresponds to RTZ handshaking. Fig 6 highlights three circuit portions. The circuit portion shown in the violet box corresponds to the synchronous QMR voter design presented in [14], with A1, B1, C1, D1 and E1 serving as the primary inputs and IX1 serving as the primary output. However, here, A1, B1, C1, D1 and E1 represent one of the dual rails of the encoded primary inputs, and IX1 is one encoded intermediate output rail.

The DRCL design method requires the construction of an original circuit and a complementary circuit to implement the logic corresponding to the dual rails of an encoded primary output. The original circuit is shown in the violet box and the complementary circuit is shown in the brown box in Fig 6. The internal outputs W1 to W5 shown in the violet box and the internal outputs N1 to N5 shown in the brown box are used to construct a part of an internal completion detector, which is depicted in the green box. In the internal completion detector, the respective pairs of the internal outputs viz. W1 and N1, W2 and N2, W3 and N3, W4 and N4, and W5 and N5 are combined using 2-input OR gates. Also, the respective dual rails of the primary inputs viz. A1 and A0, B1 and B0, C1 and C0, D1 and D0, and E1 and E0 are combined using 2-input OR gates. The outputs of all these OR gates are then combined using a C-element tree to generate the output of the internal completion detector which is denoted as ICD in Fig 6.

Upon the application of an input data, IX1 or IX0 may be produced early. For example, if C1 = D1 = E1 = 1, W1, W3 and W5 could become 1. Subsequently IX1 could assume 1 early, since this could happen regardless of the assumption of a data by A1/A0 and B1/B0. Therefore, to ensure that the production of a data or the spacer on the primary output rail X1 or X0 always happens after all the primary inputs are received and after all the internal processing is completed, an internal completion detector is necessary whose output should be considered to produce the primary output. Hence, although IX1 and IX0 are logically equivalent to X1 and X0, nevertheless, IX1 and IX0 are separately paired with ICD using 2-input C-elements to produce the encoded primary output (X1, X0). To obtain the RTO equivalent circuit of Fig 6, all the gates highlighted in red, blue and pink should be replaced by their respective gate duals i.e., the OR2, OR3, AND3, OA221 and AO221 gates should be replaced by AND2, AND3, OR3, AO221 and OA221 gates respectively. The QMR majority voter discussed in this sub-section shall henceforth be referred to as ‘SCG-QMV’ for brevity.

### 3.4. Proposed QMR majority voter

**Fig 7:**
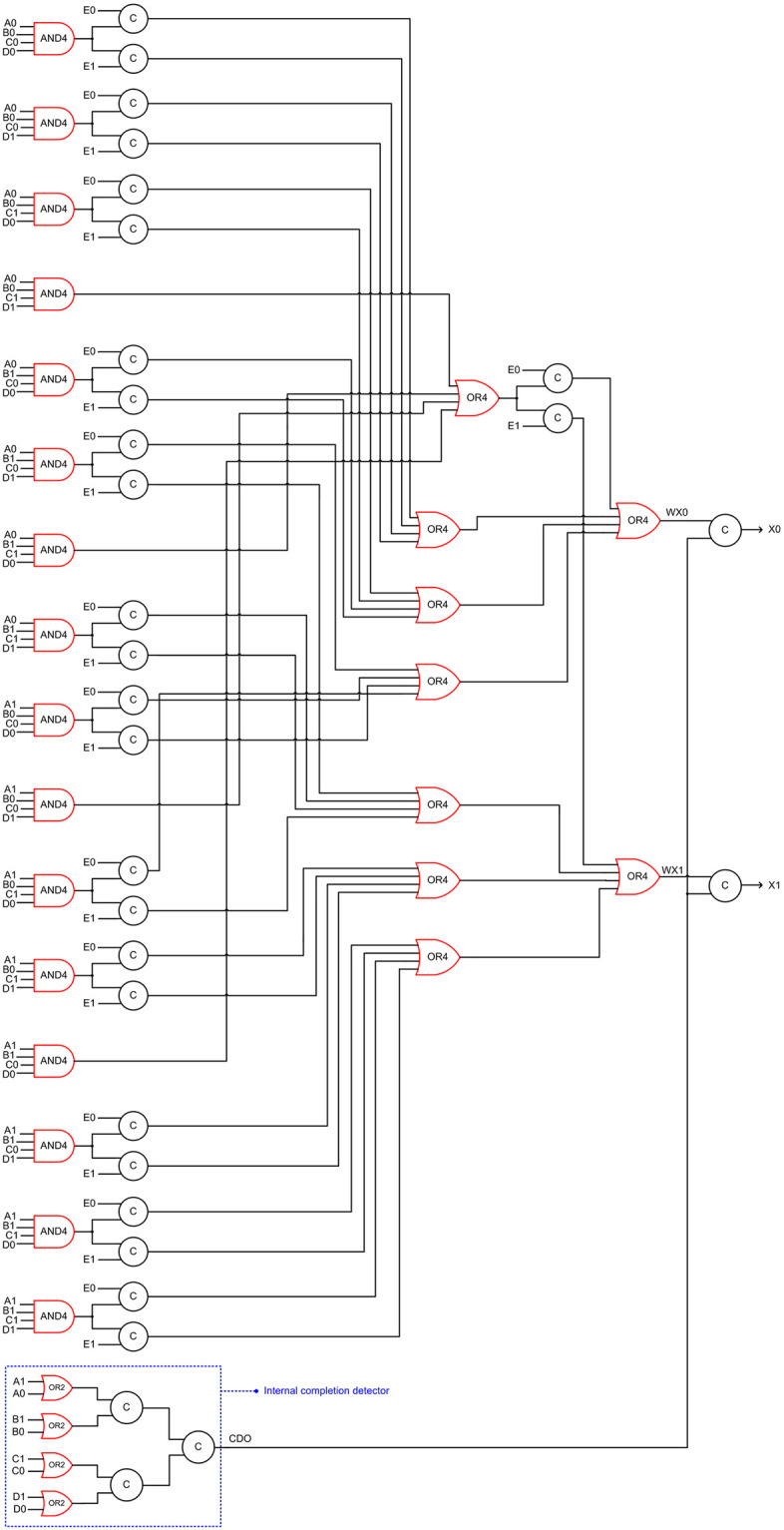
Proposed QDI QMR majority voter corresponding to RTZ handshaking. To obtain the RTO equivalent circuit, the AND4, OR4 and OR2 gates highlighted in red should be replaced by OR4, AND4 and AND2 gates respectively.

The QMR majority voters presented in sub-sections 3.2 and 3.3 are in a way our propositions because we have implemented them in a QDI asynchronous style although they are based on the synchronous QMR majority voters of [13] and [14]. Nevertheless, in this sub-section, we present our original design of a QDI QMR majority voter that is shown in Fig 7, which corresponds to RTZ handshaking. To obtain the RTO equivalent circuit, the AND and OR gates highlighted in red in Fig 7 should be replaced by their respective gate duals. In Fig 7, the arrival of a data or the spacer on E0 or E1 would always be acknowledged since E0 and E1 are connected to 2-input C-elements. However, the arrival of the spacer on all the 4-input AND gate inputs present in the first logic level may not be acknowledged in the absence of the internal completion detector. For example, if A1 = B1 = C1 = D1 = E1 = 1 in a data phase, X1 would assume 1. Following this, in the next RTZ phase, if A1 and E1 assume 0 early, X1 could assume 0 regardless of the assumption of 0 by B1, C1 and D1 if they may happen late. Therefore, to ensure the complete arrival of the spacer on A1 or A0, B1 or B0, C1 or C0, and D1 or D0 following an earlier data phase, an internal completion detector is provided which combines A1 and A0, B1 and B0, C1 and C0, and D1 and D0 using 2-input OR gates, whose outputs are synchronized using a C-element tree to produce CDO. The intermediate output (WX1, WX0) is logically equivalent to the encoded primary output (X1, X0). Nevertheless, WX1 and WX0 are synchronized with CDO using two 2-input C-elements to produce X1 and X0. This would ensure that the production of a data or the spacer on X1 or X0 would happen only after all the primary voter inputs have been received and after all the internal processing has been completed. Henceforth, we shall refer to our proposed QDI QMR majority voter using the acronym ‘P-QMV’ for brevity.

## 4. Results

Example QDI QMR circuits corresponding to RTZ and RTO handshaking were implemented by considering an early output asynchronous full adder [45] as an example function unit, like [17, 18], using a 32/28nm standard digital cell library [37]. Besides the 2-input C-element that was realized as shown in Fig 1, the rest of the gates in the cell library were utilized to implement the various QDI QMR circuits. Synopsys tools were used to design, simulate and estimate the design metrics.

**Fig 8:**
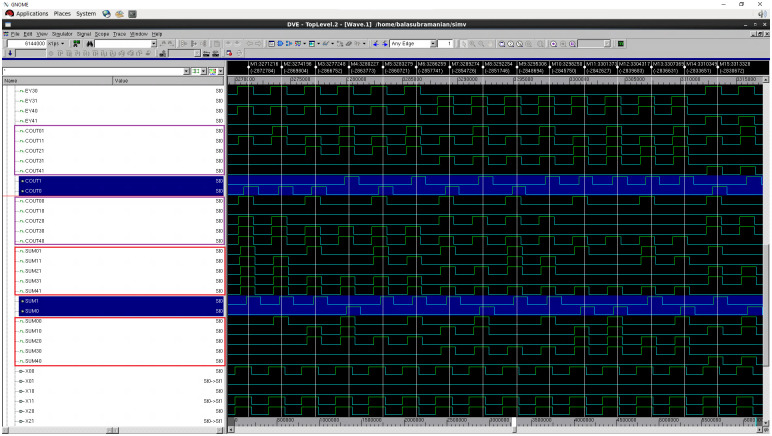
Screenshot of portion of the simulation waveform of an example QDI QMR circuit incorporating P-QMV, based on RTZ handshaking.

**Fig 9:**
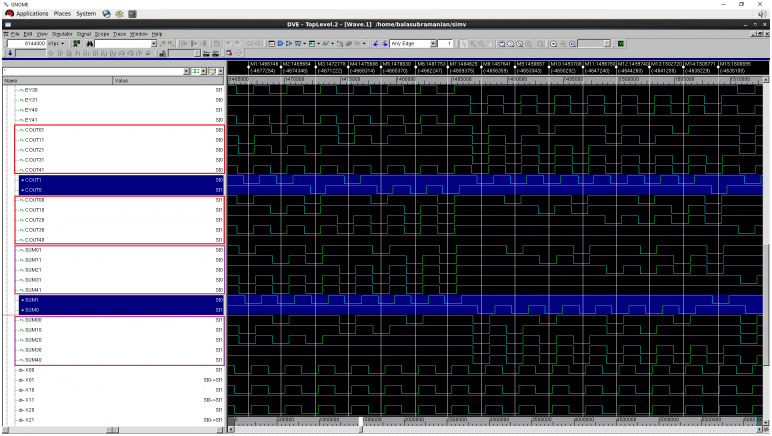
Screenshot of portion of the simulation waveform of an example QDI QMR circuit incorporating P-QMV, based on RTO handshaking.

A full adder adds three input bits and produces two output bits viz. sum and carry. The circuits of the early output full adder corresponding to RTZ and RTO handshaking are given in [17]; an interested reader may refer to the same for the details. The QMR majority voters discussed in the previous section were used along with the function units to realize the QMR circuits. The gate level simulations of all the QMR circuits incorporating the different QMR majority voters were performed by supplying all the distinct input vectors and their functionalities have been verified for both RTZ and RTO handshaking. For example, we provide screenshots of portions of simulation waveforms obtained for the QMR circuits using the proposed P-QMV based on RTZ and RTO handshaking—these are portrayed by Figs 8 and 9 respectively. Simulation waveforms similar to Figs 8 and 9 were also observed for the QDI QMR circuits utilizing the other majority voters corresponding to RTZ and RTO handshaking, validating their functionalities. The functional simulations were performed by assuming a worst-case latency of 1.5ns (i.e., a cycle time of 3ns), which is greater than the worst-case latency of SCG-QMV. The switching activity data obtained from the simulations was used to estimate the average power dissipation.

In Figs 8 and 9, (SUM01, SUM00), (SUM11, SUM10), (SUM21, SUM20), (SUM31, SUM30) and (SUM41, SUM40) represent the dual rail sum outputs of five identical full adders, with the full adders representing the function units. (COUT01, COUT00), (COUT11, COUT10), (COUT21, COUT20), (COUT31, COUT30) and (COUT41, COUT40) represent the dual rail carry outputs of the full adders. These dual rail sum and carry outputs serve as the corresponding primary inputs for the two QMR majority voters which were used along with the function units to implement the QDI QMR circuits. The primary outputs of the QDI QMR circuits are denoted by (SUM1, SUM0) and (COUT1, COUT0) in Figs 8 and 9, which are shaded in blue. The vertical markers provided in the simulation waveforms in Figs 8 and 9 are meant to guide a reader to showcase how correct majority voted primary outputs are produced when the primary inputs of the majority voters may not be the same, which is representative of single or multiple bit upsets.

In QDI circuits, the cycle time is the main timing parameter of interest. The cycle time can be considered as the critical path delay equivalent of a synchronous digital circuit which includes the set-up time. In a QDI circuit, a spacer is applied between two data inputs. Since one transaction in a QDI circuit involves the application of a data and the spacer, therefore the cycle time is the sum of the times taken for processing a data and the spacer. The cycle time determines the speed of operation of a QDI asynchronous circuit. The forward latency is the worst-case propagation delay encountered in processing the data while the reverse latency is the worst-case propagation delay encountered in processing the spacer. The cycle time is the sum of forward and reverse latencies. Commercial (synchronous) static timing analysis tools such as say, Synopsys PrimeTime was used to determine the critical path delay i.e., the forward latency and not the reverse latency. Also, the reverse latency may not be the same as the forward latency. For example, a QDI arithmetic circuit such as a QDI ripple carry adder has a data-dependent forward latency and a constant reverse latency [45], and its forward and reverse latencies are not the same. In such a scenario, the reverse latency should be estimated based on a knowledge of the gate delays encountered in the critical data path for processing the spacer. Here, since the QMR majority voters discussed are all strongly indicating, therefore the forward latency of the QMR circuits is equal to their reverse latency. Hence, the cycle time of the QDI QMR circuits is the double of the worst-case forward or reverse latency.

The design metrics estimated for the various QDI QMR circuits are given in Table 2. The split-up of average power dissipation between majority voters and others (which includes the function units, registers, and the completion detector) is also given in Table 2. In general, all the design metrics viz. cycle time, area, and average power dissipation are desired to be less. It may be noted that but for the differences in the QMR majority voters, the remaining logic of all the QDI QMR circuits are the same. This is because identical function units, registers, and completion detectors are used corresponding to RTZ and RTO handshaking. Hence, the differences between the design metrics of various QDI QMR circuits are attributable to the differences between their majority voters.

**Table 2:** Cycle time, silicon area, and averaged (total) power dissipation of various QDI QMR circuits incorporating different QMR majority voters estimated using a 32/28nm CMOS process.

QMR majority voter used	Cycle time (ns)	Area (μm^2^)	Average power dissipation (μW)		
			Voters	Others	Total
*With RTZ handshaking*					
DDIMS-QMV	2.56	736.76	38.41	295.59	334.0
Dysart-QMV	2.46	429.76	50.69	280.41	331.1
SCG-QMV	2.84	440.43	108.27	279.43	387.7
P-QMV	2.22	635.61	60.78	293.52	354.3
*With RTO handshaking*							
DDIMS-QMV	2.46	716.43	33.07	295.93	329.0
Dysart-QMV	2.44	429.76	50.67	280.73	331.4
SCG-QMV	2.82	440.43	107.60	279.60	387.2
P-QMV	2.16	649.85	59.07	296.23	355.3

Table 2 shows that the proposed P-QMV facilitates a reduction in cycle time compared to the other QMR majority voters when used to an implement a QMR circuit. This is mainly because of the fewer gates present in the critical path of P-QMV compared to its counterparts. For example, with respect to RTZ handshaking and referring to Figs 3 and 5–7, the critical path of DDIMS-QMV comprises four 2-input C-elements and two 4-input OR gates; the critical path of Dysart-QMV comprises three 2-input C-elements, three 2-input OR gates and one AO222 gate; the critical path of SCG-QMV comprises five 2-input C-elements and a 2-input OR gate; and the critical path of P-QMV comprises a 4-input AND gate, two 4-input OR gates and two 2-input C-elements. Compared to the QMR circuits employing DDIMS-QMV, Dysart-QMV and SCG-QMV, the QMR circuit employing the proposed P-QMV achieves reductions in cycle time by 13.3%, 9.8% and 21.8% respectively for RTZ handshaking. With respect to RTO handshaking, compared to the QDI QMR circuits employing DDIMS-QMV, Dysart-QMV and SCG-QMV, the QMR circuit employing P-QMV achieves reductions in cycle time by 12.2%, 11.5% and 23.4% respectively. Hence, it is inferred that the proposed P-QMV when used to realize a QDI QMR circuit would facilitate a higher speed compared to the use of other QMR majority voters.

It is also noted from Table 2 that RTO handshaking consistently facilitates a reduction in cycle time for all the QDI QMR circuits compared to RTZ handshaking. In fact, this phenomenon was found to be true for some QDI arithmetic circuits such as adders [46] and multipliers [44]. For example, the critical path of P-QMV comprises a 4-input AND gate, two 4-input OR gates and two 2-input C-elements for RTZ handshaking while the critical path of P-QMV comprises a 4-input OR gate, two 4-input AND gates and two 2-input C-elements for RTO handshaking. Based on the typical propagation delays of gates given in [37], the theoretical cycle time of P-QMV with respect to RTZ handshaking is calculated as 958ps and the theoretical cycle time of P-QMV with respect to RTO handshaking is calculated as 910ps. Since AND gates have less propagation delays compared to OR gates due to the reduced pMOS transistor stack in the former, and because there are more AND gates and less OR gates present in P-QMR for RTO handshaking compared to RTZ handshaking, therefore the former handshake protocol enables a reduced cycle time than the latter.

As seen from Table 2, the area occupancies of QDI QMR circuits slightly differ for RTZ and RTO handshaking, and this is due to the usage of dual gate types except for the C-elements. In terms of silicon area, the QDI QMR circuits incorporating Dysart-QMV occupy less area compared to their counterparts incorporating the other majority voters for both RTZ and RTO handshaking. This is because Dysart-QMV consists of fewer gates than the other QMR majority voters. For example, with respect to RTZ handshaking, Dysart-QMV occupies an area of 80.31μm^2^ while DDIMS-QMV, SCG-QMV and P-QMV occupy 233.81μm^2^, 85.65μm^2^ and 183.24μm^2^ of silicon respectively.

QDI QMR circuits comprising DDIMS-QMV and Dysart-QMV dissipate nearly the same power. However, it is important to note which majority voter dissipates less power than the rest. This is because in this work we have considered function units which required the use of just two QMR majority voters. When function units with several outputs are considered, the number of QMR majority voters would also increase proportionately and in which case the power dissipation of QMR majority voters may become significant. The split-up of average power dissipation in Table 1 shows that DDIMS-QMV dissipates less power compared to its counterparts for RTZ and RTO handshaking.

From Table 2, it is seen that the QDI QMR circuits comprising DDIMS-QMV occupy greater area compared to the QDI QMR circuits comprising the other QMR majority voters. This is because DDIMS-QMV requires more gates and so occupies more area than the other QMR majority voters. Excepting the majority voters, the rest of the logic constituting all the QDI QMR circuits are the same. However, despite the greater area occupancy of DDIMS-QMV, it dissipates less power compared to the other majority voters, as seen from Table 2. This is mainly due to the absence of an internal completion detector in DDIMS-QMV and the presence of an internal completion detector in the other QMR majority voters.

Contrary to the rest of the logic comprising a QMR majority voter, an internal completion detector would experience high switching activity. This is because, with respect to RTZ handshaking, an internal completion detector will output 1 for the application of data and output 0 for the application of the spacer during every transaction. With respect to RTO handshaking, an internal completion detector will output 1 for the application of the spacer and output 0 for the application of data during every transaction. All the gates comprising an internal completion detector will experience regular switching activity during every transactions and this will increase the total power dissipation. This explains why Dysart-QMV, SCG-QMV and P-QMV dissipate more power than DDIMS-QMV. The number of 2-input OR gates constituting the internal completion detector are 1, 10 and 4 in Dysart-QMV, SCG-QMV and P-QMV, as seen from Figs 5–7. As a result, Dysart-QMV dissipates less power than SCG-QMV and P-QMV, and P-QMV dissipates less power than SCG-QMV.

With respect to RTZ handshaking, DDIMS-QMV dissipates 24.2%, 64.5% and 36.8% less power compared to Dysart-QMV, SCG-QMV and P-QMV respectively. With respect to RTO handshaking, DDIMS-QMV dissipates 34.7%, 69.3% and 44% less power than Dysart-QMV, SCG-QMV and P-QMV respectively. Hence, from a power perspective, DDIMS-QMV is preferable, especially when many QMR majority voters may be required to implement a QDI QMR circuit/system.

**Fig 10:**
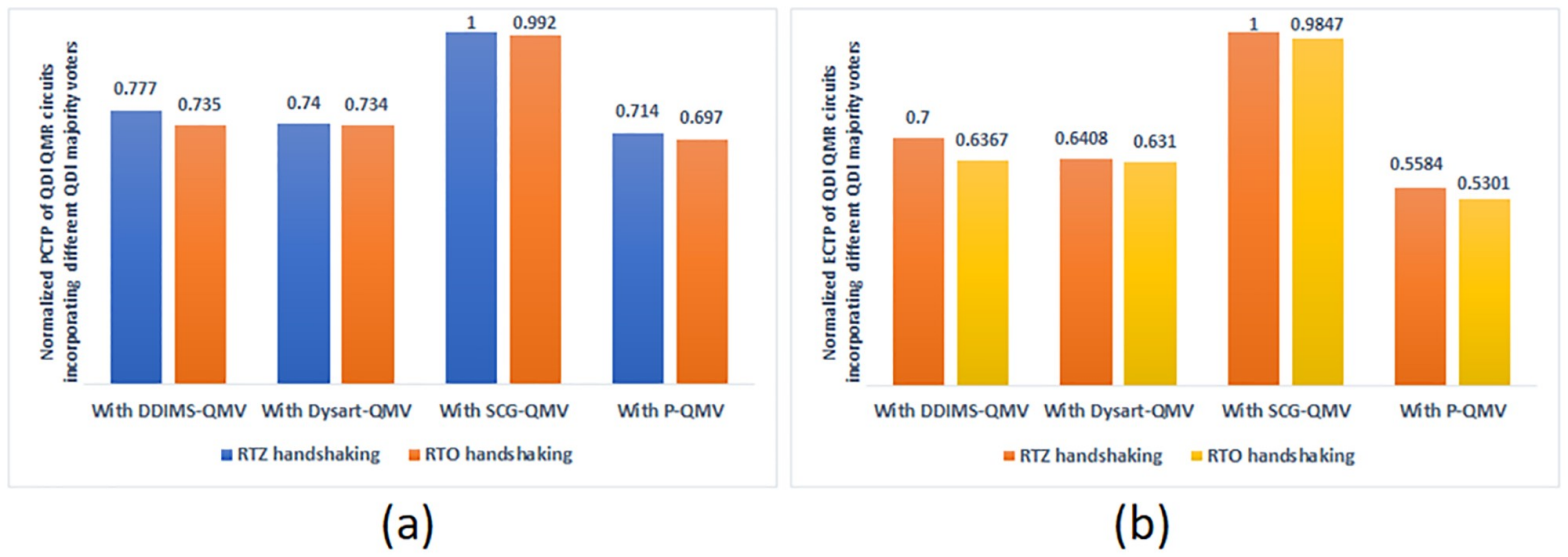
Normalized figure-of-merits of QDI QMR circuits employing different QMR majority voters corresponding to RTZ and RTO handshaking: (a) Normalized PCTP plots; and (b) Normalized ECTP plots.

In synchronous digital circuits and systems, the product of average power dissipation and critical path delay called the power-delay product (PDP) or energy, and the product of energy and critical path delay called the energy-delay product (EDP) are considered as qualitative figure-of-merits for the energy efficiency [47]. With respect to QDI asynchronous circuits, the corresponding equivalent figure-of-merits are signified by power-cycle time product (PCTP) and energy-cycle time product (ECTP). The normalized PCTP and ECTP of all the QDI QMR circuits corresponding to RTZ and RTO handshaking are plotted in Fig 10a and 10b respectively. To perform the normalization, the highest PCTP/ECTP of a QDI QMR circuit is considered as the baseline and the PCTP/ECTP of all the QMR circuits are divided by the highest PCTP/ECTP. The lesser the PCTP and the ECTP, the better is the energy efficiency of a QDI asynchronous circuit.

It is seen from Fig 10a that the QMR circuit incorporating SCG-QMV, which corresponds to RTZ handshaking, has the highest PCTP. The QMR circuit incorporating P-QMV has less PCTP compared to the QMR circuits incorporating the other majority voters for both RTZ and RTO handshaking. It is also seen that, overall, the RTO handshaking achieves a better reduction in PCTP compared to RTZ handshaking. This is mainly due to the reduced cycle time achieved for RTO handshaking compared to RTZ handshaking. The QMR circuit incorporating the proposed P-QMV, which corresponds to RTO handshaking, achieves a 5% reduction in PCTP compared to the next best QMR circuit incorporating Dysart-QMV. Thus, from the perspective of PCTP, P-QMV is preferable to the other QMR majority voters.

From Fig 10b, it is seen that the QMR circuit incorporating SCG-QMV, which corresponds to RTZ handshaking, has the highest ECTP. The QMR circuit incorporating P-QMV has less ECTP compared to the QMR circuits incorporating the other majority voters for both RTZ and RTO handshaking. It is noticed that the RTO handshaking achieves a better reduction in ECTP compared to RTZ handshaking for all the QMR circuits incorporating different QMR majority voters. This is again due to the reduced cycle time achieved for RTO handshaking compared to RTZ handshaking. The QMR circuit incorporating P-QMV, and corresponding to RTO handshaking, achieves a 16% reduction in ECTP compared to the next best QMR circuit incorporating Dysart-QMV. Therefore, from the perspective of ECTP as well, P-QMV is preferable to the other QMR majority voters.

## 5. Conclusions

Practical studies have shown that single and double bit upsets are of concern for electronic circuits and systems used in mission- and safety-critical applications. To overcome single upsets TMR can be used and to overcome double upsets QMR can be used, perhaps selectively, in the vulnerable portions of a circuit or system, according to the PMR scheme. While synchronous majority voters for TMR and QMR, and asynchronous bundled-data and QDI majority voters for TMR have been presented in the literature, asynchronous QDI majority voter designs for QMR have not yet been discussed. In this context, this article has described QDI QMR majority voters corresponding to RTZ and RTO handshaking. An analysis of different QMR majority voters when used to implement the example QDI QMR circuits shows that DDIMS-QMV and Dysart-QMV facilitate near similar low power designs, Dysart-QMV results in less area occupancy, and P-QMV is able to facilitate a high speed and energy efficient design. Overall, RTO handshaking achieves slightly better optimizations in the design metrics compared to RTZ handshaking, and the QDI QMR circuit incorporating P-QMV achieves a 11.5% reduction in cycle time, a 5% reduction in energy (i.e., PCTP), and a 16% reduction in ECTP compared to the best among the rest when considering RTO handshaking. Since speed and energy efficiency assume higher precedence than area in an electronic design, the proposed P-QMV is preferable to its counterparts.
